# A practical framework for the design of resistance exercise interventions in oncology research settings—a narrative review

**DOI:** 10.3389/fspor.2024.1418640

**Published:** 2024-12-05

**Authors:** Ciaran M. Fairman

**Affiliations:** Exercise Oncology Lab, Department of Exercise Science, Arnold School of Public Health, University of South Carolina, Columbia, SC, United States

**Keywords:** weight training, clinical trials, cancer, training principles, exercise

## Abstract

Resistance exercise (RE) has been demonstrated to result in a myriad of benefits for individuals treated for cancer, including improvements in muscle mass, strength, physical function, and quality of life. Though this has resulted in the development of recommendations for RE in cancer management from various international governing bodies, there is also increasing recognition of the need to improve the design of RE interventions in oncology. The design and execution of RE trials are notoriously complex, attempting to account for numerous cancer/treatment related symptoms/side effects. Further, the design of exercise trials in oncology also present numerous logistical challenges, particularly those that are scaled for effectiveness, where multi-site trials with numerous exercise facilities are almost a necessity. As such, this review paper highlights these considerations, and takes evidence from relevant areas (RE trials/recommendations in oncology, older adults, and other clinical populations), and provide a practical framework for consideration in the design and delivery of RE trials. Ultimately, the purpose of this framework is to provide suggestions for researchers on how to design/conduct RE trials for individuals with cancer, rather than synthesizing evidence for guidelines/recommendations on the optimal RE dose/program.

## 1 Introduction

Resistance exercise (RE) is increasingly seen as a valuable intervention in the comprehensive care of individuals treated for cancer (1–5). The results of randomized controlled trials of RE interventions in oncology have supported improvements in muscular strength, physical function, body composition and quality of life (4, 6–10). Such is the growth of the field, numerous national and international governing bodies in exercise and oncology have developed guidelines for RE and supporting its integrating into standard care practices (5, 11).

Though the development of guidelines for RE delivery in cancer is a promising development, it is also recognized that there is a clear need to enhance the body of evidence for exercise in cancer populations (2, 12). For example, the most recent guidelines from the American College of Sports Medicine outlined a limitation in the development in their guidelines, that the majority of evidence is drawn from the most common cancers (e.g., early-stage breast cancer, and prostate cancer) (5). Consequently, the ability to extend these guidelines to different tumor types, or more advanced stages of disease, is limited. Further, there are notable gaps in understanding the dose response (i.e., minimal effective dose or maximal tolerable dose) to exercise, its efficacy on specific outcomes (e.g., bone health, chemotherapy induced peripheral neuropathy and/or falls, etc.) (2, 5, 12, 13). Whilst the development and refinement of exercise guidelines in oncology represent clear forward progress for the field, it is also clear that there are numerous important gaps in where research is needed to fully understand if, and to what extent, exercise (and RE in particular) can play a role in the management of cancer.

Against this backdrop, there is also a critical conversation necessary surrounding the quality of randomized controlled trials of RE in cancer. Specifically, it has been well observed that there is a glaring lack in detail of published trials reporting clear and sufficiently comprehensive descriptions of intervention components [namely prescriptions using Frequency, Intensity, Type and Time (or FITT) characteristics], and principles of training (i.e., progression, overload, specificity) (1, 14–16). Recent systematic reviews have consistently demonstrated that these important elements remain woefully underreported in exercise oncology trials (14–16). For example, the results of a recent review indicated the 4 FITT components were fully reported in less than 60% of published trials (16). Moreover, a systematic review of RE trials alone in oncology observed only 65% reporting progression, and 76% reporting overload (1). These are important findings as without these critical elements, the field is severely hampering its own progress in developing tailored exercise guidelines for people with cancer. In fact, it is imperative that the reporting of these elements becomes a *bare minimum* requirement for trials to advance the field sufficiently in better understanding the tolerance and dose-response of exercise and contribute to future exercises.

The design and execution of RE trials in oncology also present numerous logistical challenges, particularly those that are scaled for effectiveness, where multi-site trials (or at the very least, single center trials with numerous exercise facilities) are almost a necessity. Moreover, there is consistent conversation surrounding the safety of exercise testing in oncology, along with how to best tailor/modify exercise loading in accordance with dynamic symptomology (1, 2, 17–20). As such, this paper is an attempt to bring these conversations to the forefront, and provide a practical framework for consideration in the design and delivery of RE trials in oncology. Importantly, rather than be a prescriptive approach to exercise programming, it borrows from experience with common challenges conducting RE trials in oncology, reviewing evidence from relevant areas (RE in older adults and other clinical populations) and attempts to summarize these to provide considerations for researchers in this area. Ultimately, the purpose of this framework is to provide suggestions on how to design/conduct trials testing RE interventions in oncology, rather than synthesizing evidence on the optimal RE dose/program. Lastly, it is important to underscore that many of the suggestions made in the framework should be interpreted as factors worthy of consideration for the *design of RE trials in oncology* (as opposed to recommendations for exercise prescription), rather than definitive statements based on empirical evidence of efficacy in oncology populations.

### 1.1 Outlining the framework

It's important to understand the context in which this framework can be applied. A helpful primer for this would be the conversation around the difference between “efficacy” and “effectiveness” trials in health research. *Efficacy* can broadly be defined as examining if an intervention works under ideal conditions (21, 22). *Effectiveness* then can be defined as understanding if the intervention works in real-world settings (22). Both have important roles in evaluating interventions, and the conversation of if/when these should be done in succession (i.e., efficacy prior to effectiveness) is the subject of debate. For example, researchers interested in testing the comparative efficacy of different doses of RE on specific outcomes, could certainly benefit from homogenizing the intervention approach as much as possible. Nevertheless, the outline of this paper is focused those testing *effectiveness*, for reasons (and rationale) that will become clear throughout the remaining sections.

It is rare that exercise interventions designed to examine effectiveness take place in a single site setting. In fact, it is common to at the very least have multiple exercise facilities to allow for the delivery of exercise interventions. This reality brings about important considerations as it comes to exercise selection and prescription that will be explored throughout this paper. There has been a surge in research exploring online/remote delivery of exercise interventions in cancer (23). Though important, the considerations and challenges in their design and execution warrant their own discussion. Specifically, the design of effective home-based RE interventions is notoriously complex, with additional challenges due to limited equipment, ceiling effects in increasing load/intensity, challenges with supervising safe movement patterns remotely, and fostering engagement to maintain participation (23–26). For more discussion around these challenges, readers are directed to recent literature in exercise oncology (27–37). The focus of this paper moving forward, is primarily on supervised, clinic/facility-based interventions.

## 2 Program design

**Figure 1:**
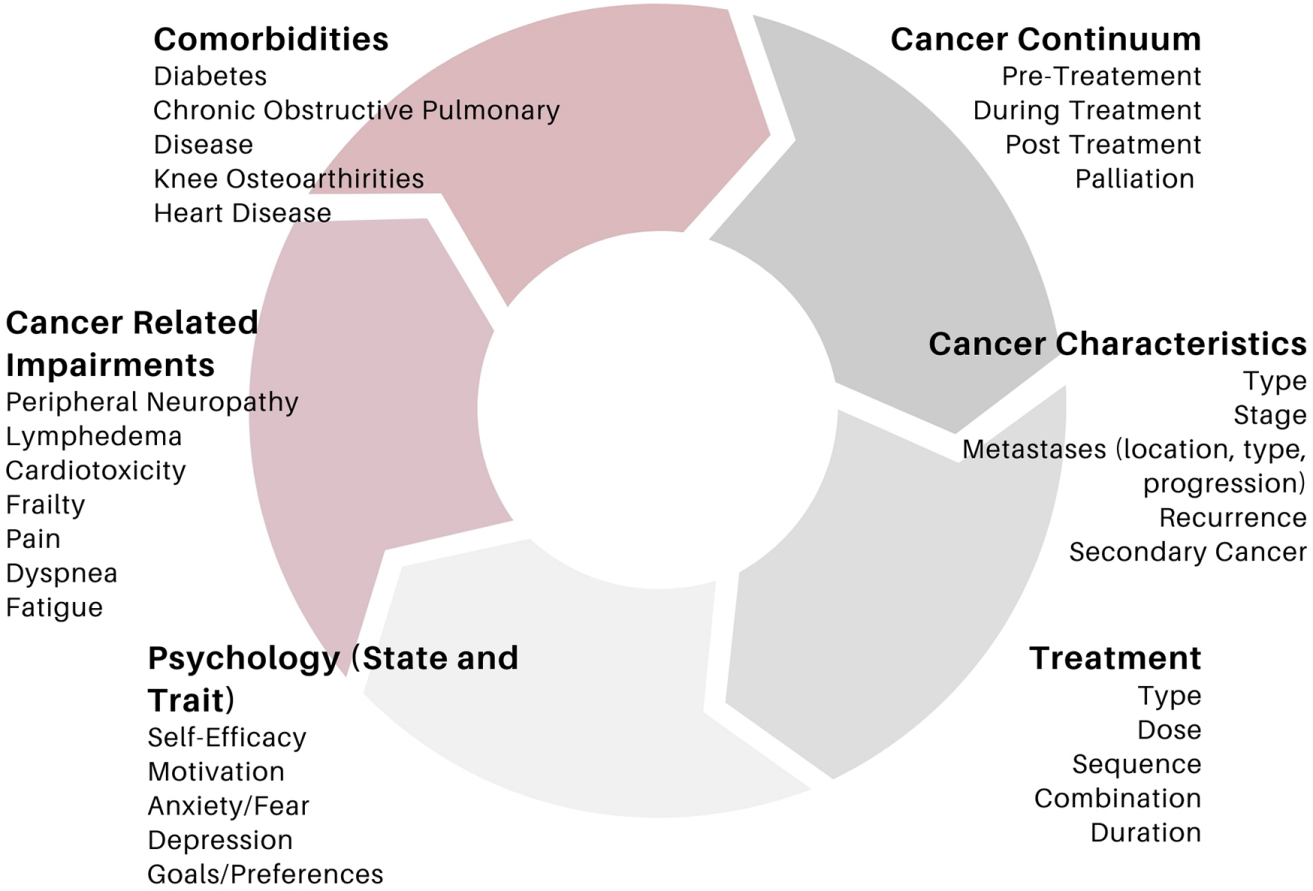
A non-exhaustive list of key considerations influencing exercise prescription and response in exercise oncology.

We and others have outlined the importance of the important of including key principles of training such as progression and overload into program design for individuals with cancer (1, 2, 12, 14, 15, 19). It is recognized that the application of these principles in oncology can be challenging for a variety of reasons. Treatment burden, symptomology, disease progression, physical limitations in addition to motivational aspects related to exercise in oncology can all impact the ability to successfully implement overload and progression in this population (13, 38, 39). Nevertheless, it is encouraged that researchers in this area strive to apply these principles as best as possible, to ensure that the full effects of exercise training can be realized. Moreover, the principles of specificity (i.e., tailoring the program to target a specific outcome), and individualization (tailoring an exercise program in accordance with an individual's baseline testing values, physical abilities and limitations (1, 2, 12, 14, 17). In oncology, there is a litany of factors that require additional consideration to ensure the program is appropriately tailored. In fact, recommendations from major governing bodies in exercise oncology place strong evidence on the need to tailor exercise programs in accordance with an individual's diagnosis, treatment type/schedule, cancer-related impairments (peripheral neuropathy, lymphedema, etc.), preferences, goals and desires (5, 11). Figure 1 outlines a non-exhaustive list of key considerations in tailoring exercise programs to individuals with cancer. Importantly, a discussion of how exercise should be tailored to each cancer-related consideration is beyond the scope of this paper. Moreover, there is insufficient evidence from the field of exercise oncology to provide detailed recommendations. Nevertheless, the complexity in interactions between these factors highlight the difficulty in designing a truly “homogenous” exercise program for individuals with cancer. As such, the following sections are outlined with these considerations in mind, in the hopes that it will foster conversation amongst researchers and practitioners alike, in how RE programs for individuals with cancer can be enhanced.

### 2.1 Determining initial loading

The principle of overload states that in order to see improvements in fitness, the body must be exposed to more stress than it is accustomed to (40). Loading refers to the amount of weight/load lifted during RE sets (41). In RE trials, the historical and most common method of determining initial exercise load is through the utilization of repetition maximum testing. This can be defined as the maximum amount of weight and individual can lift with safe and proper technique for a given repetition amount. The reps performed are typically either 1, 5, or 10. Thereafter, a submaximal percentage of this value is used to determine an initial load, for a given repetition range (i.e., 10 repetitions at 75% 1RM) (42).

Determining initial loading from %1RM remains one of the most commons methods employed in RE research (40, 43). Proponents of this method suggest that is a superior method of exercise prescription, primarily as a result of it being an objective programming strategy (43). However, the use of 1RM testing and exercise prescription based on percentages of this value, is largely rooted in sport science/strength and conditioning literature (44). In this light, loading paradigms for using %1RM typically target athletic apparently healthy populations (44). However, even in this context, it is well understood that there are large variations in the accuracy of these charts in determining repetitions performed at a given percentage (45–47). In other words, at any given percentage, there is considerable variation in the actual repetitions one can perform depending on the athlete type (strength vs. endurance), training status (trained vs. untrained), age, psychological factors and equipment used (45–47). Consequently, the utility of using %1RM to determine initial exercise load has been called into question. Perhaps most relevant to the field of exercise oncology, a case report was recently published highlighting a vertebral fracture that occurred during one repetition maximum testing in an individual with breast cancer (48). As a result, the risk-benefit ratio of 1RM testing should be carefully considered as a tool for outcome assessment and exercise prescription in exercise oncology. A great example of this is in a consensus statement published in 2022 outlining best practice recommendations for exercise testing and prescription in individuals with bone metastases (49). Herein, it is recommended that practitioners perform a risk assessment to determine exercise suitability, including garnering information on (1) bone lesion details (location, type, progression and history); (2) bone pain (rest, and/or with activity); (3) medical treatment for bone pain and (4) symptoms with bone lesions. Moreover, it is also recommended to garner additional information, including (1) detailed medical history; (2) medications/treatments; (3) history of fractures and falls; (4) bone mineral density; (5) individuals activity goals, and (6) current activity levels. Adopting pre-screening risk assessments such as the one outlined above, can help practitioners make informed choices on the risk of conducting exercise testing, relative to the value/utility of the information garnered from the test.

In addition to the above, there are several additional considerations that are worthy of discussion when considering %1RM to determine exercise loading. Firstly, there is a large time cost to doing each test. When done comprehensively, determining 1RM for a given exercise could take 15–20 min. As such, this is a considerably large time burden on participants. Secondly, it is impractical to conduct 1RM tests on every given movement pattern. For example, most common assessments in older adults/oncologic populations include a lower body 1RM (either squat or leg press) and upper body 1RM (chest press/bench press) (3, 4, 8, 10, 50–52). Consequently, the fact that the 1RM is typically only conducted on 1–2 exercises that might be included in an exercise program, limits its practical use in determining exercise intensity. Perhaps most importantly, the rapid neuromuscular adaptations that occur with RE in previously untrained individuals is well established. Improved firing rate and coordination of motor unit recruitment, reduced inhibition of contraction, familiarity with movement patterns can all contribute to rapid and substantial increases in strength in the first few weeks of RE (53–55). As a result, this increase in strength also renders the utility of prescribing exercise load of %1RM from baseline values rather useless. In fact, it could be argued that using a given% from baseline values to prescribe a training load just a few weeks in previously untrained individuals could be underdosing the exercise (wherein the individuals 1RM would have increased too).

Whilst using %1RM to determine exercise loading still has a lot of meaningful applications in RE research, it's certainly worth considering alternatives to this approach that consider the logistical (time, participant burden), safety and practical constraints (participant effort, limited number of exercises assessed, training status reducing accuracy) of delivering RE prescription in a variety of settings in oncology.

#### 2.1.1 Alternatives

A practical alternative to 1RM testing is the use of familiarization phases. The familiarization phase recognizes the complexity and challenges of assisting (commonly) relatively inexperienced individuals beginning a RE program. As such, the phase employs a systematic approach to coaching different movements, identifying appropriate exercises and loads, ensuring safe technique, and anchoring procedures for various scales (37, 56). Further, the use of familiarization phases allows for a gradual build up towards appropriate loading for a specific repetition range. For example, many recommendations for older adults and individuals with cancer suggest 1–3 sets of 8–12 repetitions for RE. Inherently, this will involve some experimentation in the first 1–2 weeks in attempting to determine the appropriate loading for each exercise. Moreover, given that governing bodies recommend 6–8 exercises targeting major muscle groups, it's unlikely that participants/trainers will be able to determine the appropriate load for all exercises in a single session. Consequently, allowing several days for familiarization allows for prioritizing 2–4 exercises in any given session, gradually building on these across the coming weeks. Additionally, the use of a familiarization phase overcomes some of the limitations of relying solely on %1RM to determine load, by being able to dynamically adjust load to fit an RM target as practice and neurological adaptations contribute to strength adaptations early in the intervention.

As an alternative to %1RM loading for prescription and progression, the use of “repetitions in reserve” (RIR) has emerged as a viable alternative to determine exercise loading to for RE (57, 58). RIR is a subjective scale that estimates how many perceived repetitions an individual has remaining following the completion of a set (Table 1) (59). Several versions of the scale exist, though commonly, the scale is scored from 0 to 10, with the number provided corresponding to perceived repetitions remaining. For example, an RIR of 2 would indicate that the perceived repetitions remaining was 2. The scale has been demonstrated to be valid and reliable and has been utilized in several trials of RE in apparently healthy individuals (59, 60). It is worth noting however, that the scale has not been empirically testing within the field of exercise oncology. Further, the accuracy and reliability of RIR increases with familiarity with the scale, and when estimating repetitions closer to failure (i.e., 1–3 RIR) (57, 61). Additionally, simply having target repetition ranges can also serve as a useful tool for loading. For example, if the target range is 10–12, anything over 12 reps would necessitate a load increase, whereas a failure to achieve 10 repetitions could necessitate a load decrease. Nevertheless, the use of RIR/repetition ranges to determine exercise intensity could be a practical/feasible option to consider in the design of RE trials in oncology.

**Table 1:** Repetitions in reserve.

RIR	Estimated reps. remaining
0	0, momentary failure
1	1
2	2
3	3
4	4
5	5
6	6
7	7
8	8
9	9
10 +	10 +

#### 2.1.2 Additional loading considerations

A detailed outline of all the possible combinations of loading paradigms and progressions is beyond the scope of this paper. However, readers are referred to several positions stands and resources for program design for older adults, individuals with cancer, and clinical populations (11, 41, 42). Nevertheless, individuals with cancer experience a variety of cancer/treatment related impairments that require considerations in the program design worthy of note (5, 12, 62). One consideration with regards to this, is whether the RE program is designed to accommodate the impairment (i.e., configure the RE program such that the impairment isn't a barrier to exercise participation), or to challenge the impairment (i.e., the impairment is something that is understood to be targetable though exercise to slow/stop/reveres the impairment).

Dyspnea (shortness of breath) is a particularly common occurrence in individuals with lung cancer (63, 64). This dyspnea often becomes a barrier to exercise, where “air hunger” can occur with exercise. As such, this results in the avoidance of activity and subsequent deconditioning (65–67). A consideration for this could be to accommodate the impairment through manipulation of rest periods (26, 37, 68). By strategically incorporating extra rest during exercise, this may increase tolerance to exercise, which could result in greater adaptations in from the intervention. Therefore, specifically designing exercise sessions in a way that accommodates, instead of exacerbates, dyspnea might increase participation in exercise and ultimately, support improvements in clinically relevant outcomes.

Contrastingly to the above, reductions in gait speed, physical function, balance, and bone health can increase the risk of falls and fractures in individuals treated for cancer (69, 70). These impairments might require a specific emphasis on balance training, with the inclusion of power exercises performed at higher velocities. In fact, performing exercises at higher velocities can served to recruit high threshold, type II muscle fibers, and may improve force generating capacities greater than those performed at slower velocities (41). This could contribute to a better ability to perform activities of daily living, and a protective effect on fall risk. Consequently, designing the RE program specifically with a goal of challenging impairments (i.e., muscular strength, power, physical function, etc.), could result in greater improvements in clinically relevant patient outcomes, and long-term prognosis (Table 2).

**Table 2:** Loading considerations for different program foci.

	Cancer relevant outcomes	Sets	Repetitions	Frequency	Intensity	
Power	Rate of force development; gait speed; physical function; fall risk	1–3	3–6	2–3 days/week	40–60% 1RM	4–5 RIR
Strength^a^	ADLs; muscular strength; fall risk; physical function; body composition	1–3	1–6		70–85% 1RM	2–3 RIR
Hypertrophy^a^	ADLs; muscular strength; fall risk; physical function; body composition; endurance	1–3	8–12+		65–80% 1RM	2–3 RIR
Endurance	ADL's, endurance	1–3	10–15		50–70% 1RM	2–4 RIR

^a^ It is recognized that individuals who are resistance training naïve, will likely see improvements in strength at a wide range of intensities, including those listed under “hypertrophy”. Nevertheless, it's likely that to see continuous improvements, progression towards higher intensities/load may be required.

### 2.2 Exercise selection

Traditionally, exercise oncology trials have used standardized methods of delivering RE programming (1, 62). The results of a recent systematic review of RCTs using RE in oncology revealed that over 50% of studies have used employed similar protocols (1). Typically, these are machine based exercises, targeting major muscle groups of the upper and lower body. Practically, this is typically the following: leg press, leg extension, leg curl, chest press, seated row, shoulder press. There are several advantages to this approach. Intuitively, employing standardized exercise prescription across different studies allows for the potential comparison of effects between studies. Moreover, standardization of the exercise selection allows for reduced variability that could confound results, improved quality control, and increased internal validity (71).

Despite its advantages, employing a standardized, machine-based approach in exercise oncology is not without its limitations. Firstly, though important for standardization, it could be reasonably suggested that performing the same 6–8 exercises for months could contribute to boredom and/or staleness, impacting the likelihood the of continuing exercise. It is not uncommon that large RCT's in exercise oncology are performed at different sites. This can often mean that different facilities have different machines, or even variations within the same machine's angles/moment arms/loading mechanisms. Moreover, exercise prescription in oncology regular requires modifications to the dose in accordance with symptomology (fatigue, nausea, energy etc.). Thus, inherently there is *already* a degree of variability in the exercise prescription, where the program is not inherently standardized between participants. Importantly, several researchers are making the claim to acknowledge this as a reality and normalize this within exercise oncology research.

Secondly, it is well established that the long-term uptake of RE behavior is notoriously poor (72, 73). Whilst several hypotheses for the poor uptake of long-term RT behavior exist, there are a couple that are of relevance. Interventions that employ rigid exercise prescriptions, using the same 6–8 exercises on identical machines, limit the ability of participants to replicate this protocol when transitioning to independent maintenance of activity, particularly those at different facilities (i.e., where the participants facility might not have the same machines used in a study). Additionally, the use of rigid exercise prescription approaches using predominantly machine-based exercises, violates the key training principle of individualization, in addition to the critical and overlooked principle of variation (systematic process of altering training variables/exercise selection of to keep the training stimulus novel and avoid staleness) (41, 74). The latter is particularly important, given the close link between exercise challenge (or novelty), enjoyment and variation and long-term adherence. As such, employing the same exercise selection over a considerable period (i.e., 12–52 weeks) as is common in RCTs in oncology may promote staleness, reducing the motivation/desire to continue participation. Rooted in established theories of behavior change such as the self-determination theory and social cognitive theory, exercise programs focused on fostering perceptions of variety and challenge could facilitate greater long-term maintenance of RE behaviors (75, 76).

Individuals with cancer are often older individuals, commonly presenting with comorbidities, previous injuries and physical limitations that require modification to exercise sessions, including exercise selection (63, 77, 78). Though this has traditionally been poorly documented, more recent publications have made efforts to normalize this (79, 80). Essentially, altering exercises between participants is already relatively common in the field. Taken collectively, there is ample evidence to suggest that adopting a more flexible approach to RE prescription in exercise oncology trials could serve to (1) better adhere to key principles of training such as individualization and variability, and (2) serve to potentially enhance the likelihood of long-term adoption and maintenance of RE behaviors.

An alternative to using static exercise prescriptions, predominantly utilizing machine-based exercises, would be to employ a prescription and progression model based on body movements. In many of the recommendations for RE, multi-joint compound movements (i.e., squat, deadlift) are recommended as (1) they recruit a large amount of muscle tissue, providing a strong stimulus for adaptation and (2) large compound movements often mirror movements conducted in activities of daily living (i.e., getting out of a chair, sitting down into a car, picking up laundry etc.) (41, 42, 81, 82). As such, there is a strong rationale to shift towards a prescription model that focuses on the movement pattern, rather than a specific exercise. Many individuals with cancer present with vastly different physical limitations, training history and cancer symptomology. Consequently, incorporating a squatting pattern (rather than a specific exercise), allows to better individualize the program, where one individual may be able to complete a goblet squat, and another may have difficulty standing up out of a chair unsupported. Table 3 outlines an example of how variations of exercises can be employed to target specific muscle groups. Herein, the same movement pattern can be targeted, but the stimulus from can be individualized to different physical capabilities.

**Table 3:** Sample exercise variation template.

	Variation 1	Variation 2	Variation 3	Variation 4	Variation 5	Variation 6	Variation 7
Hip hinge		BW hinge with dowel	KB/DB RDL	Trapbar deadlift	Increase load	Barbell deadlift	Single leg RDL
Squat	Mobility exercises	Supported BW squat	STS	Weighted STS	BW squat	Goblet squat	Barbell squat
Horizontal push	Wall push up	Incline Push up	BW push up	DB bench press	BB bench press	Increase load	
Horizontal pull	ROM exercises	TRX row	Seated row	Bent over DB row	Bent over BB row	Increase load	
Vertical push	ROM exercises	OH BW press	Landmine press	DB/KB shoulder press	Increase load		
Vertical pull	ROM exercises	Close grip pulldowns	Lateral pulldowns	Increase load	Pull ups		
Core	Palloff press	Increase load	Tandem stance Pallof press	Pallof press with rotation	Side plank	Side plank row	Farmer carries

BW, bodyweight; DB: dumbbell; KB, kettlebell; OH, overhead press; RDL, Romanian deadlift; STS, sit to stand; TRX, TRX suspension trainer.

### 2.2.1 Exercise order

General principles of exercise order are rooted in the sequencing of exercises to minimize the impact of fatigue on exercise performance and maximize the stress/stimulus from a given session. In exercise oncology trials, it's rare to see RE interventions have >3 workouts per week. In this manner, generally the recommendation would be to employ a model of full-body workouts, targeting all major muscle groups in each session. In this model, general principles for exercise order would be to perform large multi-joint, compound movements before small/single-joint exercises (e.g., Push up before triceps pulldown, squats before leg extensions, etc.). Additionally, training muscular power requires an individual to complete movements at higher velocities (56, 83). Consequently, when incorporating strength, power and hypertrophy in the same training session, it is recommended to complete power exercises first, such that fatigue from prior exercises don't negatively impact the ability to train appropriately for power development (41, 42). Likewise, strength requires an individual to perform exercises at relatively high loads, so it is recommended that this is performed prior to higher-volume, fatiguing exercises focused on hypertrophy. The incorporation of exercises targeting power should be incorporated before strength hypertrophy exercises and in order from most to least complex, to minimize the impact of fatigue on performance (41). Table 4 provides an example of exercise order based on programmatic foci. Of note, the exercise examples and order in Table 4 refer to the primary “working” exercises of an exercise session. Traditional RE warm ups include short (3–10 min) bouts of cardiovascular exercise, coupled with light stretching prior to beginning this session. Particularly if beginning a program with complex multi-joint strength/power based movements, it is worth noting that the addition of a dynamic warm-up (e.g., similar movements without load, or smaller isolation type movements) may be worth considering to appropriately prime these movements (84).

**Table 4:** Examples of exercise order for specific foci.

	Exercise	Sets	Reps	Weight	Reps in reserve
Balance	Semi tandem stance	2	30 s		n/a
	Flamingo stand	3	8		n/a
Power^a^	Chair stand	4	2		4–5
	MB bounce pass	4	3		4–5
Movement capacity	Floor transfer	2	BW		n/a
	BW hinge with dowel^b^	2	10		3–4
	Lunge^c^	3	10		2–3
	Bent over row	3	10		2–3
	Incline push ups	3	10		2–3
	Shoulder press	3	10		2–3

^a^ Focus on speed of movement, explosive during concentric action.

^b^ Focus on movement pattern, hinging at hips with neutral spine.

^c^ Focus on increasing pain free ROM, appropriate knee joint angles and stance width.

Ultimately, it is uncommon that individuals with cancer experience one impairment (i.e., loss of balance without strength, or low bone mineral density without physical function impairments) (85). Consequently, it may very well be that the next frontier of exercise oncology RE research employs multi-component interventions, using a variety of loading and exercise selection paradigms to simultaneously target multiple impairments (i.e., balance, power and strength) (86). As such, careful consideration should go into strategic sequence of specific program foci, and the order of exercises and across an intervention duration.

### 2.3 Progression

The principle of progression in RE states that for continuous improvements to occur, the stimuli must be systematically altered to provide greater stress over time (41, 42). Practically, this is achieved by altering the volume, intensity and/or complexity of exercise (41). Identifying systematic approaches to progressing RE loads safely and efficiently remains the topic of ongoing conversation (42, 87, 88). Nevertheless, overarching principles are that progression should be implemented from low difficulty/intensity to higher difficulty/intensity in accordance with the principle of individualization, consideration of comorbidities/symptoms (i.e., knee osteoarthritis or pain etc.), and individual training experience (41). There are a number of ways in which the stress of exercise can be progressed, including (1) the resistance (weight/load); (2) altering sets and repetitions; (3) altering speed of repetitions; (4) altering rest periods; (5) altering support (double stance, single stance, unstable surface) and (6) a combination of any of these listed (41, 89). A review of the strengths, limitations, and applications of each of these concepts is beyond the scope of this review. In this light, readers are directed to other excellent reviews/positions stands on RE program design (41, 83). Further, there are very few trials specifically comparing different progression models of RE in older adults, clinical populations, or individuals with cancer. As such, given the added complexity of a cancer diagnosis on fatigue/symptomology, there is unlikely to be a one-size fits all approach to RE progression. However, outlined below are several ways in which exercise oncology researchers might consider incorporating these approaches.

General progression models have been recommended an employed in older adults and cancer, including the “2 for 2” rule. This rule suggests that an individual should increase the resistance (load/weight) when they can perform two or more repetitions over their target number in the last set of an exercise for two consecutive workouts. For example, if someone is aiming for 8–12 reps in a set and they can do 14 reps comfortably for two workouts in a row, this is an indication to increase the weight. This rule helps ensure progressive overload, which is key for muscle growth and strength gains. Suggestions of increases in volume are commonly between 2.5% and 10%, though these should be interpreted with caution given the substantial variation in physical capabilities and exercise tolerance in individuals with cancer (41, 42). Using the 2 for 2 rule could provide initial signal that the load can be progressed, where the next load selected is sufficiently high to be challenging, but remains safe and still falls within the target repetition range. It's also important to note that, as previously mentioned, there are a variety of factors (symptom burden, disease progression, fatigue, pain, etc.) that can impact an individual's ability to tolerate exercise, let alone progress load from session to session. As such, it is also worth utilizing systems that provide guidance on reducing the load. For example, failure to achieve the lower end of a target rep range of 8–12 outlined above could necessitate a reduction in volume to avoid excessive fatigue or too quick of a progression. It is unlikely that the field of exercise oncology will progress to a point where we have specific guidance by which when and how much to progress/regress exercise difficulty from one session to the next for each tumor type/cancer setting. Nevertheless, the principle of monitoring and adjusting load is paramount, and how much to change load can also be determined by using the same principles of achieving specific rep ranges or RIR.

An important and often overlooked aspect of progression, is altering the exercise selection/level of support. In Table 3, above, we have outlined examples of exercise variations falling within a given movement pattern. Further, this same emphasis on movement patterns allows for progression of exercises *within* an individual. In line with recommendations for RE with older adults, the exercise can be progressed by altering the level of support. Essentially, individuals with low mobility, function and strength may require additional support for a given exercise (using a suspension trainer, or a partner to get out of a chair in a sit-to-stand exercise). Once sufficient strength has been achieved, the exercise can be progressed by removing support, moving towards an unsupported sit-to-stand or a bodyweight squat. Continued progress can be achieved by moving towards a loaded movement (goblet squat or barbell back squat), or a single-stance movement (lunge, Bulgarian split squat).

RE progression can be achieved through a variety of methods. Though more research is warranted to understand how to strategically progress programs to optimize outcomes of interest. The focus of progression should firstly be on safety, management of exercise tolerance/workload, and recovery. Additionally, this requires an additional layer of consideration, particularly during active treatment, where fatigue, pain, nausea, low energy and other symptoms can fluctuate dramatically, impacting exercise tolerance and recover. As such, it's worth considering monitoring these factors in exercise interventions to have plans in place to account (outlined in section 5).

Whilst there is a clear need for the field of exercise oncology to apply and report the principle of progression more often and in more clear detail (1, 14–17, 90), it is also important to note that this may not always be possible/a priority. For example, there may be times where the burden of cancer/treatment is so large, that progression of exercise volume/intensity may not be possible (91, 92). In these cases, it is worth considering utilizing regression protocols (i.e., autoregulation, outlined in section 2.5) to accommodate fluctuations in symptom burden, stress and exercise tolerance. Moreover, in long-term exercise programs (months-years), continuous progression may not be attainable/the primary focus. In these programs, maintaining exercise volume could also be beneficial for long-term adoption and maintenance of RE behaviors (75, 76).

### 2.4 Variation

It is not uncommon for trials of RE in oncology to last 6 months and beyond. Here, there principle of variation is exceptionally important. As alluded to above, alterations in training variables and the training stress are imperative for continued progress, but also to avoid staleness with exercise participation. As such, the systematic manipulation of volume, intensity, and acute program variables (i.e., sets, repetitions, exercise selection etc.) can be importantly to ensure continued progress and engagement with RE behavior. This variation (typically called periodization, though this concept has been questioned recently) can also assist with managing rest and recovery from exercise (2, 93–95). Typical models include beginning with high volume/low intensity of training, gradually progressing towards lower volume and higher intensity (focusing on strength and power) (93). This achieved through training phases specifically focused on distinct physiological outcomes. For example, 8-weeks could be spent with the program focused on hypertrophy, and a subsequent 6-weeks on strength. This process is generally referred to as linear periodization.

Other models of variation have been proposed, most commonly advocating for a nonlinear approach, whereby these volume and intensity are manipulated on a more frequent basis, typically weekly (i.e., week, 1 hypertrophy; week 2, strength; week 3, power) or even daily (i.e., Monday, hypertrophy; Wednesday, strength; Friday, power) (94, 96, 97). Advocates for this approach propose that it allows to target all components more frequently, and allowing for more engagement through variety, and potentially improved rest and recovery. Importantly, there is no conclusive evidence demonstrating superiority of either a linear or nonlinear approach to program variation. Nevertheless, what is clear is that the variation itself is an essential component of optimizing outcomes, where periodized programs consistently demonstrate superior benefits in strength and function compared to on-periodized programs (98). As such, researchers designing RE trials should consider the systematic design of longer-term interventions to include elements of variation that includes the manipulation of training volume and intensity (12).

### 2.5 Accommodating variations in stress and symptomology

**Figure 2:**
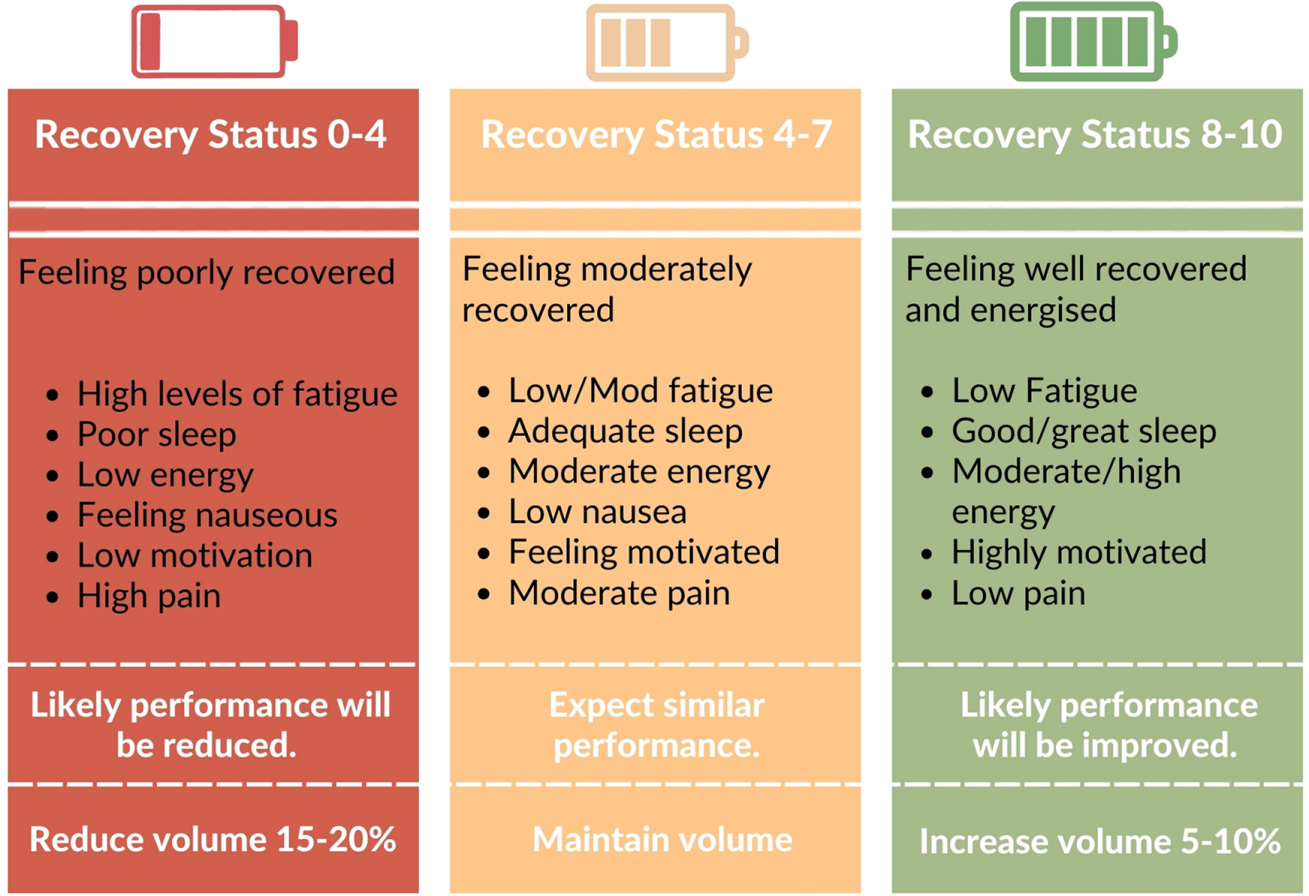
Hypothetical trajectories of physiological and psychological stress across cycles of chemotherapy. Symptoms fluctuate daily/weekly, whilst also compounding over time. The figure also highlights the inverse relationship between physiological and psychological stress, and readiness to train (ability to participate in and respond to a given training stimulus).

In addition to the heterogeneity that exists in the cancer population (tumor type/stage, treatment characteristics, time along the cancer continuum, etc), there is also considerable heterogeneity in the incidence, magnitude and severity of symptoms experienced by individuals treated for cancer (63, 99–101). It is well documented that fatigue, sleep disruption, mood disturbance, pain, energy, and appetite changes are common occurrences with cancer treatments (63, 99, 102–107). However, it is also documented that these side-effects/symptoms can fluctuate sometimes drastically, both within/across days during and after treatment (108–111). Further, it has also been documented that symptomology may “compound” across time with treatment, such that the symptom burden become greatest as treatment (chemotherapy in particular) continues (112, 113). Consequently, it is logical to hypothesize that when physiological and psychological stress is at its highest, the desire and physiological readiness (i.e., “readiness to train”) to participate in and respond to a given straining stress is at its lowest (Figure 2) (2).

The fluctuations in physiological and psychological wellbeing across cancer treatments influencing one's readiness to train has resulted in several calls to modify the training stress in accordance with the variations in fatigue and symptomology (2, 12, 114). The concept of autoregulation (monitoring and adjusting the training stimuli in accordance with fluctuations in daily readiness to train) is relatively new to exercise oncology, with only one trial completed to date incorporating it in their intervention (114). However, whilst there is currently no empirical consensus of which method is optimal for systematically modifying training stress, there is strong agreement that symptomology should be monitored during exercise, and that the training stimuli should be adapted in some way to accommodate fluctuations in symptoms (5, 11, 12, 114, 115).

**Figure 3:**
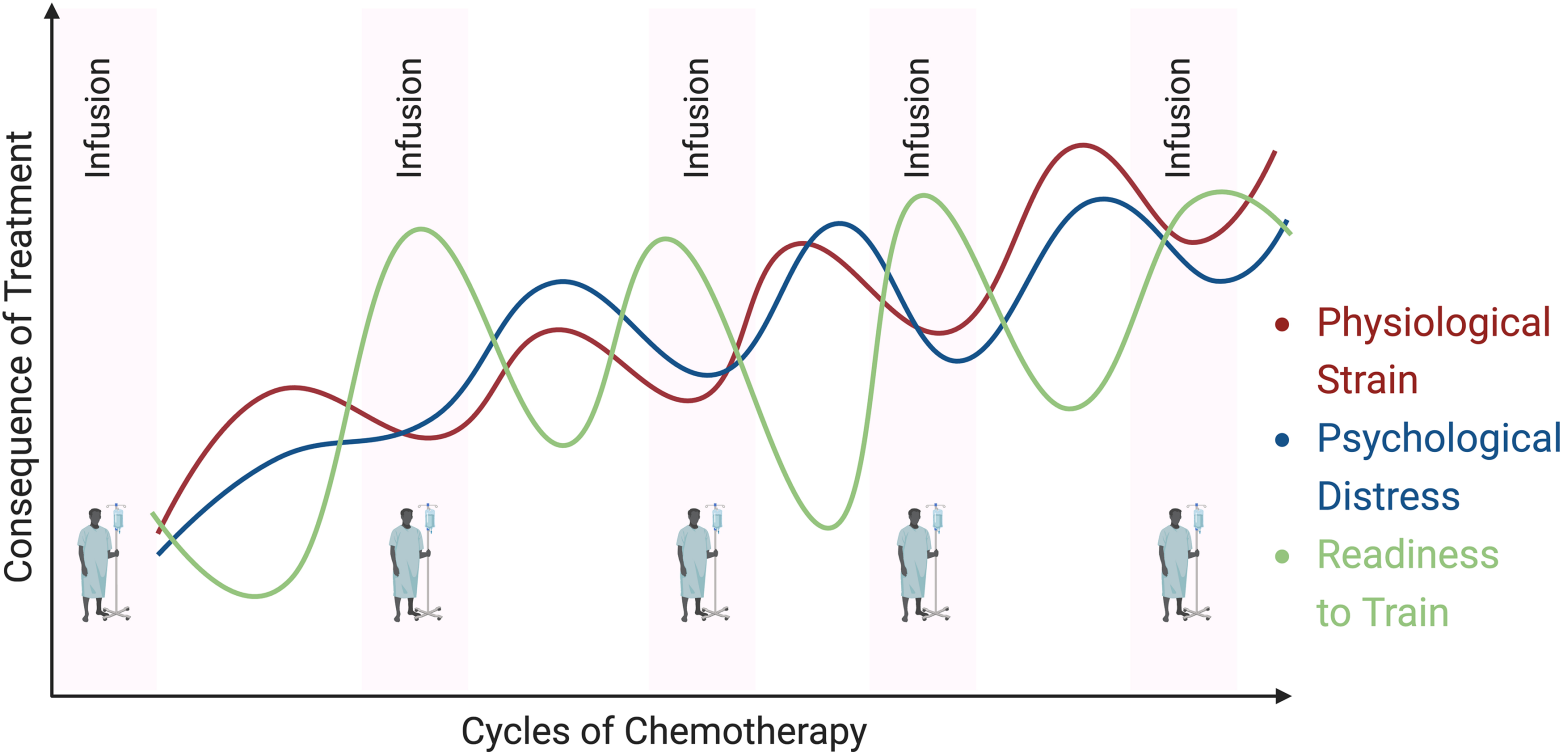
Example of scale employed for autoregulation. Individuals provide a recovery score based on perceptions of symptom burden. This could then inform changes in RE stimulus to match readiness to train. Though exercise volume is used in this example, it is understood that there are innumerous ways RE variables could be manipulated to alter training stress.

One approach to apply autoregulation would be to use a composite of common symptoms experienced by individuals with cancer to determine a subjective readiness to train. For example, pain, sleep, nausea, fatigue and motivation are all understood to fluctuate during treatment. Based on perceived burden of these symptoms, a “recovery status” score could be provided, which would indicate potential performance for a session. This score could be used to adjust the training variables and subsequent training stimuli, to match readiness to train (Figure 3). There are a variety of subjective and objective methods of autoregulation currently being investigated in RE interventions (116, 117). It's worth noting that there is currently no direct evidence in exercise oncology that compared the relative effectiveness of autoregulated vs. standardized RE. Nevertheless, the investigation of systematic approaches to accommodating fluctuations in symptoms and motivation in exercise oncology is warranted to better understand how exercise prescription can best be individualized and tailored to optimize outcomes whilst managing exercise tolerance.

### 2.6 Reporting dose and adverse events

One of the primary limitations in developing tailored exercise recommendations in oncology is a lack of information on the “dose” of exercise received (39). Exercise trials typically report adherence to exercise, most commonly achieved through reporting attendance (yes or no) to an exercise session. Though attendance at exercise sessions is important, this information does very little to capture the tolerance to exercise, particularly in circumstances where exercise modifications as a consequence of symptoms is a common occurrence (13, 38, 39). To address this, novel metrics have been proposed to better capture the dose of RE prescribed, in addition to the dose of RE actually completed. Using volume of RE (sets × reps × weight; or sets × reps) the exercise-relative dose intensity (ExRDI), is calculated as a ratio of actual volume achieved to total volume prescribed (whereby in individual achieving a volume of 7500 lbs compared to a prescribed 10,000 lbs, the ExRDI would be 75%) (13). The adoption of metrics such as ExRDI expand on adherence to allow for a more precise quantification of exercise dose and tolerance, to support the development of tailored exercise recommendations.

As the volume of exercise oncology trials rapidly expands, it has become apparent that there is a glaring lack of understanding of the potential risk/harms (79, 80). Recently, a framework has been published to improve the monitoring and reporting of exercise-related adverse events in oncology (79). Briefly, this framework outlines procedures for (1) monitoring the occurrence of adverse events; (2) recording the type, severity, causality of adverse events and their impact on the intervention; (3) reviewing the causality, relationship to the intervention and whether or not they were anticipated; and (4) reporting frequencies, rates and details of all-cause and exercise-related adverse events (79). Researchers are strongly encouraged to adopt rigorous and systematic approaches to documenting and reporting adverse events to better support the development of risk:benefit assessments of exercise in oncology.

## 3 Limitations

It's important to underscore several limitations with this framework. Firstly, as mentioned above, it is recognized that this framework introduces variability into the design of RE interventions that could confound results, particularly if the desired goal is testing the efficacy of an intervention. This is an important limitation that should be considered within the larger goal of designing research questions and subsequent training programs. However, as mentioned above, the primary focus of this paper is on supervised interventions, focused on effectiveness (namely, can it work in a community setting). As such, those aiming to rigorously evaluate the efficacy of specific doses of exercise may require different consideration than those outlines above. Secondly, it is also recognized that some of the suggestions outlined in this framework do not have direct evidence from exercise oncology to support their use (i.e., the use of RIR to manage load). Rather, this information is taken from a collective body of literature including RE research in apparently healthy individuals, recommendations for RE in older adults, clinical populations, and individuals with cancer. Additionally, the information outlined in the framework is also taken from the author's anecdotal experience conducting randomized controlled trials of RE in individuals with cancer and clinical populations. Consequently, the framework should be interpreted with caution and understood to be an attempt to offer guidance to others designing RE clinical trials, rather than specific recommendations for RE implementation based on evidence of efficacy. Lastly, the framework focuses primarily on cancers impacting adults (i.e., >18 years). The author recognizes the limitation of excluding adolescent and young adult individuals with cancer from this paper. However, this was intentional in that the program design and challenges of developing frameworks in this population are highly nuanced and would be best served with a framework specifically tailored to them.

## 4 Conclusion

As the field of exercise oncology continues to grow, there are worthy conversations around enhancing the rigor, quality and creativity in the design of RE interventions for individuals with cancer. This paper summarizes considerations as they relate to (1) determining initial loading; (2) exercise selection; (3) applying the principle of progression; (4) applying the principle of variation; (5) accommodating variations stress and symptomology; and (6) reporting training dose and adverse events. The hope is that these suggestions offer guidance for researchers seeking to design RE interventions specifically tailored for individuals diagnosed with cancer.
